# The influence of schooling on cognitive screening test in the elderly

**DOI:** 10.1590/S1980-57642008DN10100008

**Published:** 2007

**Authors:** Gustavo Christofoletti, Merlyn Mércia Oliani, Florindo Stella, Sebastião Gobbi, Lílian Teresa Bucken Gobbi

**Affiliations:** 1 Graduate program in Ciências da Motricidade – Institute of Biosciences, UNESP, campus of Rio Claro/SP.; 2 Clinic of Geriatric Psychiatry, Medical Sciences School (FCM), UNICAMP, CRUESP – Academic Cooperation Program.

**Keywords:** neuropsychological tests, cognition, elderly, education, testes neuropsicológicos, cognição, idosos, educação

## Abstract

**Introduction:**

Tests for screening cognitive functions are gaining importance with the increasing incidence and prevalence of demential syndromes. For our elderly population, the challenge is to develop neuropsychological tests independent from the influence of educational level.

**Objective:**

To compare the influence of education on the elderly with or without cognitive decline, on the Brief Cognitive Screening Battery (BCSB).

**Methods:**

We studied 176 elderly people: 60 with cognitive decline (aged 73.6±9.3 years and with 5.7±0.7 years of education) and 116 without cognitive impairments (aged 73.4±0.6 years and with 5.6±0.5 years of education). The BCSB was applied in all subjects. The data were submitted to descriptive statistics and analyzed by Independent Student test with 95% confidence intervals.

**Results:**

The data showed that the BCSB is an appropriate battery for identifying cognitive status in normal elderly individuals, as well as cognitive decline in our elderly sample. The BCSB items were not significantly influenced by schooling years, making this test favorable for different groups characterized by illiterate individuals, as well as by those with low or high levels of formal education.

**Conclusion:**

The BCSB proved to be a useful cognitive screening test for old people with or without cognitive decline independent of their educational level.

## Abstract (pt)

**Introdução:**

Instrumentos capazes de aferir as funções cognitivas vêm ganhando importância como aumento na incidência e prevalência de quadros demenciais. O desafio consiste em criar instrumentos que não sofram influência dos níveis de escolaridade.

**Objetivo:**

Analisar comparativamente a influência do nível de escolaridade na Bateria Breve de Rastreio Cognitivo (BBRC) em idosos com e sem declínio cognitivo.

**Métodos:**

Participaram do estudo 176 idosos, sendo 60 (idade de 73,6±9,3 anos e escolaridade de 5,7±0,7 anos) com declínio cognitivo e 116 (idade de 73,4±0,6 anos e escolaridade de 5,6±0,5 anos) sem queixas cognitivas. Foram aplicados a BBRC em todos os sujeitos. O tratamento dos dados consistiu na aplicação de estatística descritiva e do teste t–Student independente, admitindo-se um intervalo de confiança de 95%.

**Resultados:**

Os dados indicaram que a BBRC consiste em um instrumento apropriado para a identificação da condição cognitiva em idosos normais, bem como, para confirmar declínio cognitivo em idosos comprometidos do nosso meio. Os itens da BBRC não sofreram influência significativa dos anos de escolaridade, fator que favorece sua aplicação em diferentes grupos, compostos por indivíduos iletrados, bem como por sujeitos com pouca ou elevada escolaridade.

**Conclusão:**

A BBRC mostrou-se um instrumento útil para rastreio cognitivo em idosos, independentemente de serem ou não letrados.

The performance of elderly people in daily activities reflects their cognitive organization and, possibly, the integrity of their cortical function. Memory, language, reasoning, executive functions, perception, capability of recognition, and praxis are cognitive activities achieved by distinct and integrated cortical areas. Disorders in these areas commonly indicate the beginning of a demential process^1,2^.

Cognitive screening instruments are important for clinics, and their application involves specific knowledge and procedures in accordance with the nature of each test and with the characteristics of the population to be examined^3^.

Educational level represents a factor that influences the performance on such tests. For illiterate individuals, for example, some cognitive tests require an adjustment of scores, or a cutoff point according to the intellectual requirements that their several tasks demand^4,5^.

The Mini-Mental State Examination (MMSE)^6^, a widely used cognitive screening test, is significantly influenced by educational level^7^. Developed by Nitrini et al.^8,9^, the Brief Cognitive Screening Battery (BCSB) is useful to identify cognitive functions of both literate and illiterate individuals, as its execution is little influenced by education^10^. This study aimed to compare the influence of education of elderly people, with or without cognitive decline, on BCSB scores.

## Methods

In this transversal design study, we researched 176 male and female, elderly people, with mean age of 73.4 ±7.3 years and education of 5.6±0.4 years. Subjects were divided into two groups:

a) 116 residents of Rio Claro, SP, cognitively preserved; and
b) 60 institutionalized individuals with cognitive decline confirmed by clinical evaluation, living in two nursing homes in the Rio Claro/SP region.

A random group of cognitively preserved elderly from the community was evaluated using the MMSE and the BCSB to assess cognitive condition, along with the Pfeffer Questionnaire of Functional Activities^11^ to assess normal performance on instrumental daily activities. Inclusion criteria in this group were based on expected cognition for normal cognitive status suggested by Brucki et al.^7^ and Nitrini et al.^8,9^, and on normal performance according to the Pfeffer Questionnaire. Subjects with inferior cognition for their respective schooling levels, or those with impairment on instrumental daily activities, as well as individuals with neuropsychiatric conditions, or using psychotropics were excluded from this group.

The institutionalized selection of cognitively impaired subjects was established based on history of cognitive decline and clinical evaluation. These procedures were carried out by psychiatrists from the respective institutions, based on the ICD-10 Classification of Mental and Behavioral Disorders^12^. The MMSE and the BSCB were also applied to corroborate cognitive impairment, while the Pfeffer Questionnaire was applied to confirm mental condition associated to cognitive decline. Subjects visually or auditory handicapped, and those with neuropsychiatric conditions incompatible with the proposed tasks were excluded from this group.

The BCSB^8,9^, a straightforward instrument for evaluating cognitive functions in elderly people, consists of the presentation of a sheet of paper with 10 common figures (shoe, spoon, hair-comb, tree, turtle, key, airplane, house, book and bucket). Each of the objects must be identified and named by the individual (identification/naming), and then immediately recalled without prior information that the objects had to be memorized (incidental memory). Subsequently, the figures are presented to the individuals, who are requested to memorize and recall them (immediate memory). The figures are then presented again, in order to be memorized, and evoked (learning). The figures are presented one more time to the subjects for memorizing (delayed memory), but where subjects must evoke them later on, after subsequent interference with the semantic verbal fluency test^13^ (animals/minute) and the clock drawing test^14^. Finally, the 10 figures are shown together with another 10 distractors, where the individual has to recognize those figures initially presented (recognition). Administering the procedures of the BCSB takes approximately 7 to 10 minutes.

The descriptive statistics (mean and standard deviation) and the independent Student-t test were used for data quantitative analysis. This test allowed an intragroup comparative analysis (important to observe whether the educational level influenced BCSB items) and an intergroup analysis (to reveal evidence of accuracy of this instrument for identifying cognitive decline). Confidence intervals of 95% were established.

The study was previously approved by the Research Ethics Committee of the State University of São Paulo – UNESP, Rio Claro-SP. A free and informed consent term was devised according to resolution 196/96 of the Brazilian Ministry of Health, and all subjects gave written consent to participate in this research.

## Results

The sample of 176 subjects was classified into two groups: 116 elderly people residing in the city of Rio Claro, SP – Brazil, cognitively preserved (mean age of 73.4 ±0.6 years and education of 5.6±0.5 years), and 60 institutionalized elderly people with cognitive decline (mean age of 73.6±9.3 years and education of 5.7±0.7 years). By the independent Student-t test, there was no significant difference regarding age and educational level between the groups (p>0.05).

Table 1 shows the BCSB scores of subjects with cognitive decline, and those cognitively preserved, according to educational level. Results from the intergroup analysis of BCSB are described in Table 2.

**Table 1:** Descriptive statistics (mean and standard deviation) of age and BCSB according to educational levels of: a) 60 institutionalized elderly subjects with cognitive decline; and b) 116 elderly subjects without cognitive decline.

	Education (in years)				
	0	1 to 4	5 to 8	9 to 11	> 11
**a) Institutionalized elderly subjects with cognitive decline**					
Sample size	7	31	9	3	10
Age	81.3±3.6	72.0±1.7	75.5±2.0	68.3±7.3	72.8±2.9
BCSB					
Identification/naming	7.7±1.1	8.8±0.4	9.5±0.4	10	10
Incidental memory	2.7±0.9	2.8±0.4	4.1±0.6	3.0±1.7	3.2±0.7
Immediate memory	4.1±0.9	3.2±0.4	4.1±0.6	3.3±2.0	5.0±0.8
Learning	4.8±0.8	3.4±0.4	4.,3±0.7	3.7±2.1	5.2±1.2
Verbal fluency test	7.1±2.1	6.9±1.0	10.5±1.7	8.6±1.7	10.6±1.7
Clock-drawing Test	1.7±0.6	2.3±0.5	4.3±1.0	4.3±2.0	7.0±1.1
Delayed recall	3.8±1.1	2.1±0.5	2.3±1.0	3.0±1.7	4.4±1.1
Recognition	5.8±1.3	5.3±0.6	6.4±1.0	5.7±1.4	7.8±1.1
**b) Elderly subjects without cognitive decline**					
Sample size	10	63	18	11	15
Age	77.7±2.1	73.1±0.7	70.8±1.6	75.3±2.3	74.2±1.3
BCSB					
Identification/naming	10	9.9±0,1	10	9.5±0.5	10
Incidental memory	5.2±0.4	5.4±0.2	5.3±0.4	5.0±0.5	5.4±0.4
Immediate memory	7.2±0.4	7.4±0.2	7.1±0.4	7.3±0.7	7.5±0.3
Learning	7.6±0.7	7.5±0.2	7.9±0.5	7.5±0.6	7.8±0.3
Verbal fluency test	15.1±2.0	13.7±0.5	15.3±1.2	16.0±1.3	19.6±1.2
Clock-drawing test	6.5±1.1	7.5±0.3	8.5±0.4	9.0±0.1	8.9±0.4
Delayed recall	7.6±0.6	7.6±0.2	7.9±0.4	7.7±0.7	8.0±0.4
Recognition	9.8±0.1	9.5±0.2	9.4±0.3	9.8±0.1	9.9±0.1

BCSB, brief cognitive screening battery.

**Table 2:** Intergroup difference (F and significance level) related to BCSB.

	Education (in years)				
	0	1 to 4	5 to 8	9 to 11	> 11
**BCSB**					
Identification/Naming	27.9	29.7*	10.9	1.4	**
Incidental Memory	6.8*	8.7*	0.4	1.4	6.1*
Immediate Memory	2.9*	7.2*	0.1*	0.7*	13.4*
Learning	0.1*	12.1*	1.6*	1.2*	31.1
Verbal Fluency Test	1.1*	12.8*	3.2*	7.6*	11.0*
Clock-Drawing Test	21.1*	66.9*	6.3*	14.3	33.1
Delayed Recall	0.2*	9.2*	0.2*	0.7*	0.6*
Recognition	7.4*	0.3*	9.9*	15.3	8.4

BCSB, brief cognitive screening battery;

* Statistically significant difference (p<0.05);

** According to independent student-t test, it was not possible to carry out the "identification/naming" in subjects with an educational level over 11 years due to their same scores.

On the independent Student-t test, the intragroup analysis demonstrated a significant influence of educational level on the clock-drawing test in both groups (p<0.05). However, in this population other items of the BCSB were little influenced by educational level (p> 0.05), although a small trend for increased scores related to higher educational levels was observed, mainly in subjects with cognitive decline.

**Graph 1:**
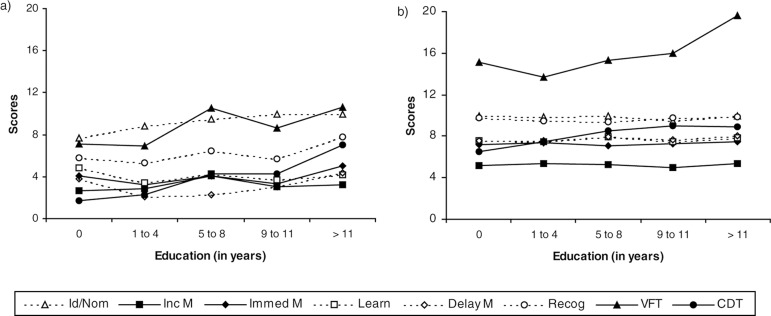
Intragroup results of BCSB items according to the educational level of: A) 60 elderly subjects with cognitive decline; and B) 116 elderly subjects without cognitive decline.

The verbal fluency test presented an unusual finding reported in Graph 1, characterized by non-uniform pattern of word generation according to schooling years. When we then compared word generation for a specific education level with another immediately successive level (for example: illiterates versus 1 to 4 schooling years) we did not always observe significant influence of schooling on verbal fluency (p>0.05). However, when we compared subjects with low education against those with 5 or more schooling years we found a significance influence of formal education on word generation (p<0.05). Results from the intragroup analysis of BCSB are depicted in Graph 1.

## Discussion

Our study confirmed the low influence of schooling years on word generation in both groups composed of both cognitively preserved and cognitively impaired individuals, as expected. On the other hand, we observed an important influence of this variable on scores of the clock drawing test in both groups. Regarding the verbal fluency test, our results presented peculiarities outlined below.

In a previous Brazilian study, Caramelli et al.^15^ demonstrated that the verbal fluency test (animals/minute) is a useful instrument for screening cognitive decline, mainly in mild Alzheimer´s disease patients, when specific cutoff scores are adjusted according to progressive years of formal education. Brucki et al.^16^ found significant influence of schooling years on verbal fluency scores, and also recommended specific cut-off points for progressive years of formal education. Another study^17^ further corroborated the influence of schooling on verbal fluency performance.

Concerning the generation of semantic category, there are distinct suggestions for specific cut-off scores related to progressive schooling levels, proposed by several Brazilian authors. These differences could be partially explained by sociocultural characteristics strongly influencing language abilities. Although many elderly people in Brazil were classified as illiterate, this situation may not be homogeneous. Thus, the communication media accessible to all population segments, exposing these people to the literate world, probably minimizes the deficiency of knowledge acquisition at school. Nevertheless, school attendance can represent an important influence on the domain of metaknowledge demanded by language tests such as verbal fluency, among other language evaluation instruments^17^.

Regarding word generation, our results have some peculiarities in comparison with previous reports^15,16^. In contrast to some studies, the present work did not display a uniform improvement in verbal fluency according to successive schooling years. This non-uniformity may have occurred in our sample due to the variability in the number of subjects belonging to respective schooling levels. Besides, when we compared subjects with low levels of schooling to those with high schooling levels, we found a significant influence of formal education on verbal fluency. Concerning clock drawing, the present study reasserts previous results regarding the influence of formal education on cognitive performance in this test^18^. On the other hand, the BSCB items were not significantly influenced by schooling years, and this characteristic is likely based on the structure and nature of this battery.

It is appropriate to emphasize the lack of evidence of any significant difference between the cognitively preserved group and the group with cognitive impairment regarding age and educational level (p>0.05). Indeed, a similarity between these variables was observed.

Our study confirms the hypothesis by Nitrini et al.^8^ concerning the low influence of literacy, commonly associated with schooling level, on items of the BCSB. In addition, the present findings provided evidence for the benefit of BCSB for people living in our setting, characterized by social, demographic, and cultural heterogeneity.

This neuropsychological battery is efficient for cognitive impairment assessment of elderly people independent of their literacy performance, especially in illiterate patients who present mild Alzheimer’s disease and whose cognitive impairment results from expected memory decline.

In conclusion:

a) The BCSB was able to evaluate cognitive functions in all subjects from both groups, cognitively preserved and with cognitive impairment, respectively.
b) Educational levels had a small influence on the BCSB; this aspect is favorable with regards to cognitive screening for diagnosis of dementia in the elderly population living in our country, characterized by illiteracy or low education.
c) The BCSB does not require adjustment of the cutoff point scores related to educational levels;
d) The BCSB is a straightforward and useful neuropsychological battery that can be performed easily by illiterate elderly people, as the instrument consists of several simple procedures for screening cognitive impairment and monitoring its process of decline.
